# Supplementary material for: Dynamic glucose enhanced imaging using direct water saturation
Source: Magn Reson Med. 2025 Mar 17;94(1):15–27. doi: 10.1002/mrm.30447 (PMC12021318; doi:10.1002/mrm.30447)
Supplement: Supplementary file 1 — TABLE S1. Exclusion criteria for the study. [file MRM-94-15-s001.docx]

**Supporting Information**

**Dynamic Glucose Enhanced Imaging using Direct Water Saturation**

**Linda Knutsson^1,2,3^ ⏐Nirbhay N. Yadav^1,4^⏐ Sajad Mohammed Ali^3^ ⏐ David Olayinka Kamson^2,5^⏐ Eleni Demetriou^1,4^ ⏐ Anina Seidemo^6^ ⏐ Lindsay Blair^2^ ⏐ Doris D. Lin^4^⏐ John Laterra^2,5,7,8^ ⏐**

**Peter C. M. van Zijl^1,4,9^**

^1^F.M. Kirby Research Center for Functional Brain Imaging, Kennedy Krieger Institute, Baltimore, MD, US

^2^Department of Neurology, Johns Hopkins University School of Medicine, Baltimore, MD, US

^3^Department of Medical Radiation Physics, Lund University, Lund, Sweden

^4^Russell H. Morgan Department of Radiology and Radiological Science, Johns Hopkins University School of Medicine, Baltimore, MD, US

^5^Department of Oncology, Johns Hopkins University School of Medicine, Baltimore, MD, US

^6^Diagnostic Radiology, Department of Clinical Sciences, Lund University, Lund, Sweden

^7^Hugo W. Moser Research Institute at Kennedy Krieger, Baltimore, MD, US

^8^Department of Neuroscience, Johns Hopkins University School of Medicine, Baltimore, MD, US

^9^Department of Biomedical Engineering, Johns Hopkins University School of Medicine, Baltimore, MD, US

**Correspondence**

Linda Knutsson, PhD

F.M. Kirby Research Center

Kennedy Krieger Institute

716 N. Broadway

Baltimore, MD, 21205, United States

E-mail: [lknutss1@jhu.edu](mailto:knutsson@kennedykrieger.org)

**Supporting Table S1.** Exclusion Criteria for the study

| Exclusion Criteria |
| --- |
| Age <18 |
| Unwilling to participate in the study |
| Not capably to understand or sign inform consent |
| Presence of any ferromagnetic implant (cardiac pacemakers, aneurysm clip, etc.) |
| Pregnancy |
| Claustrophobia or anxiety disorder |
| Diabetes mellitus (self-reported or hemoglobin A1c ≥6.5%) |
| eGFR documented within the last 2 weeks less than 60 mL/min/1.73 m^2^ |
| Sickle cell disease |
| Blood iron deficiency (hemoglobin concentration <12 g/dL, hematocrit <35%) |
| Multiple myeloma |
| Solid organ transplant |
| History of severe hepatic disease |
| Liver transplant or pending liver transplant |
